# A nova teoria da ciência de Thomas Kuhn e o livro não finalizado A Pluralidade dos Mundos

**DOI:** 10.1590/S0104-59702026000100043

**Published:** 2026-10-19

**Authors:** Fernanda Schiavo Nogueira

**Affiliations:** i Doutoranda, Universidade Federal de Minas Gerais. Belo Horizonte – MG – Brasil. fernandaschiavonogueira@gmail.com

**Figure:**
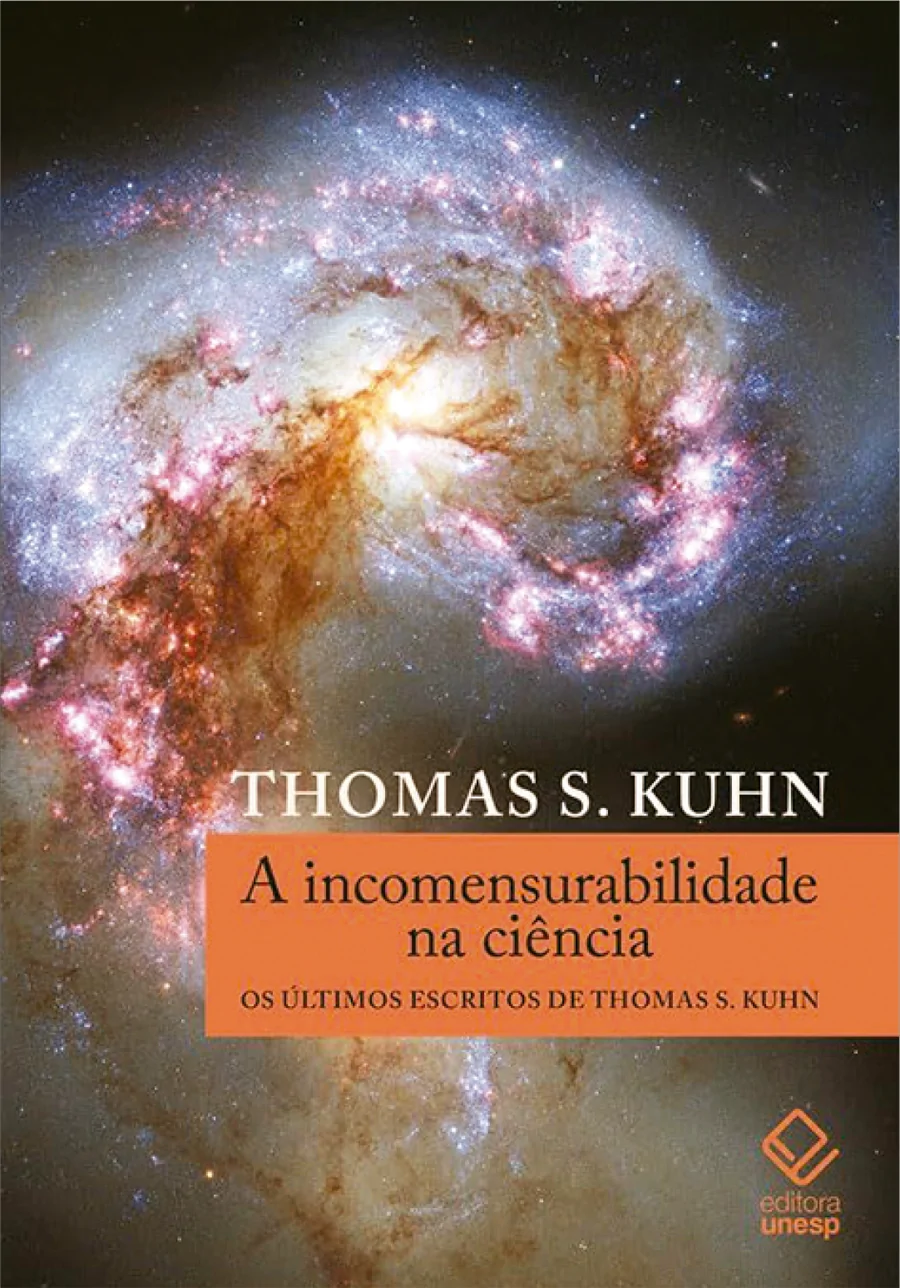

O ano de 2022 marcaria o fim da espera de mais de 30 anos dos leitores de Thomas Kuhn. À época, a Universidade de Chicago publicou, em inglês, os textos que ele escreveu para a constituição do livro no qual estaria a nova teoria da ciência prometida pelo autor. Dois anos depois, a Unesp lançou tradução em português da obra então intitulada *A incomensurabilidade na ciência*. Todos os textos apresentados na coletânea foram compilados por Bojana Mladenovic, professora de filosofia do Williams College. *A incomensurabilidade na ciência* visava reconstruir os contornos globais das ideias abarcadas no projeto de livro interrompido pela morte de Kuhn, considerado pela organizadora “a palavra final” sobre os impasses originados em *A estrutura das revoluções científicas* (Mladenovic, 2024, p.21).

*A incomensurabilidade na ciência* demandou a investigação de um vasto acervo de fontes e a organizadora decidiu incluir, no volume, materiais não publicados. São eles: dois artigos, três apresentações orais realizadas pelo autor, os cinco primeiros capítulos completos da obra inacabada, o rascunho do sexto e anotações difusas dos restantes. Os registros mobilizados condensariam as mudanças no entendimento da constituição da ciência e estariam destinados a ser reunidos em livro então em andamento. Contudo, todos permaneceram na forma de esboços, uma vez que foram compreendidos por Kuhn como notas ainda muito preliminares da nova abordagem em desenvolvimento.

Em “Thomas Kuhn’s legacy for the historiography of science” [O legado de Thomas Kuhn na historiografia da ciência], Mauro Lúcio Leitão Condé (2023) chama atenção para a inspiração decisiva e abrangente exercida por *A estrutura das revoluções científicas*, obra que influenciou da vida cotidiana aos mais diferentes campos do conhecimento. Segundo Condé (2023, p.122), “o livro teve o impacto mais significativo de todos os tempos” com mais de um milhão de exemplares vendidos. Contudo, o sucesso sem antecedentes foi acompanhado de muitas polêmicas sobre a uniformização do pensamento provocada pelo paradigma e sobre as descontinuidades abruptas causadas pela revolução e a incomensurabilidade global. Diante das dificuldades, Kuhn sempre procurou esclarecer o que para ele eram problemas de entendimento da leitura, embora, gradualmente, tenha identificado a insuficiência das correções pontuais que fizera ao longo de sua carreira.

Desde o posfácio da segunda edição de *A estrutura*, Kuhn iniciou o trabalho de reformulação das propostas de interpretação defendidas. Todavia, o tratamento dado à constituição do conhecimento da ciência deveria ganhar revisão ainda mais profunda. Assim, o autor, gradativamente, sentiu a necessidade de elaborar uma nova teoria da ciência a ser reunida em livro futuro, obra infelizmente não terminada. Das mudanças implementadas, Kuhn modificou dois conceitos-pilares (revolução, incomensurabilidade), abandonou outros (*gestalt*, paradigma,) e criou novos (léxico, especiação do conhecimento). O foco da preocupação da obra planejada residiria na principal fonte dos problemas enfrentados por *A estrutura*: a ideia de incomensurabilidade. Foram incansáveis as tentativas do autor voltadas para reduzir a intensidade da falta de comunicação entre linguagens diferentes (o que explicaria o título dado à coletânea: *A incomensurabilidade na ciência*).

Cada cientista, quando socializado, aprenderia a linguagem manipulada pela comunidade de falantes estruturada em taxonomias utilizadas no entendimento da natureza: o léxico. Qualquer léxico criaria instrumentos de intervenção que permitiriam o conhecimento de universos diferentes de fenômenos, capazes de garantir o acesso aos mais variados mundos (o que justificaria como a obra inacabada de Kuhn foi intitulada: *A pluralidade dos mundos*). A escolha de qual taxonomia seria a predominantemente aprendida não resultava da correspondência entre linguagem/natureza: a mais efetiva representação em palavras do funcionamento do mundo. O que motivaria a adesão seria a capacidade de satisfazer demandas e necessidades gerais decorrentes da busca por melhores resultados na manipulação dos fenômenos.

Como cada especialidade do saber apresentaria taxonomia específica, as ramificações surgidas em cada disciplina ou as inovações introduzidas descreveriam mudanças de intensidades diferentes na linguagem praticada. As alterações implantadas permitiriam ao conhecimento existente evoluir a novos patamares ao longo do tempo. Cada transformação sofrida pela taxonomia adotada, apta a introduzir novas especialidades, seria operada pela revolução. O termo utilizado evocaria convencionalmente a eclosão de descontinuidades abruptas entre o antigo e o novo conhecimento, marcadas pela falta de comunicação.

Como a evolução foi adotada como mecanismo de desenvolvimento predominante na ciência, o poder de transformação tradicionalmente vinculado à revolução foi atenuado. Do corpo mais antigo de conhecimento (a ascendência cognitiva comum), o saber seria desdobrado em variadas outras especialidades, disciplinas sucessoras consideradas com alcance expandido e ideias revisadas (fenômeno da especiação). A ideia de evolução, semelhante à biologia (embora operada pela revolução), possibilitaria a transmissão de heranças do saber predecessor às gerações futuras, tais como: metodologias, problemas e soluções basilares. Logo, o conhecimento não mais seria totalmente perdido, mas ampliado, corrigido e adequado às novas circunstâncias.

A regularidade dos intercâmbios estabelecidos entre a disciplina irradiadora e as novas especialidades diminuiria a frequência e o grau de intensidade das barreiras existentes à plena troca de conhecimentos. No lugar da impossibilidade de entendimento, existiriam problemas de linguagem capazes de dificultar a compreensão imediata, mas não impedir radicalmente a comunicação. A falta completa de superposição das taxonomias ocorreria apenas em situações raras, quando os saberes compartilhados pertencessem a culturas ou épocas muito distantes.

Cada declaração com pretensão ao conhecimento não possibilitaria a compreensão plena dos interlocutores, caso recorressem à mera tradução. O procedimento, quando aplicado, sempre fomentaria a proliferação de ideias discrepantes. A superação das dificuldades de entendimento derivaria da proficiência em novos léxicos, ou seja, a capacidade de responder em cada uma das linguagens aprendidas (o multilinguismo). Dominar taxonomias diferentes seria questão tributada à atuação de estruturas cognitivas inatas dos seres humanos, as mesmas responsáveis pelo aprendizado do idioma materno.

O ineditismo dos documentos reunidos em *A incomensurabilidade da ciência* contribui decisivamente para o melhor entendimento da trajetória intelectual de Kuhn. Havia pistas pontuais e isoladas dos direcionamentos tomados pelo pensamento do autor desde o posfácio de *A estrutura*. A coletânea, no entanto, revela de forma mais clara quais rumos as ideias de Kuhn tomaram em seguida. As principais mudanças incorporadas foram a atuação central da linguagem, a atenuação das descontinuidades provocadas pela revolução e a defesa da incomensurabilidade local. No entanto, as novidades introduzidas não conduziram o autor ao rompimento absoluto com duas das concepções mais controversas: revolução e incomensurabilidade (embora tenham sido reformuladas).

*A incomensurabilidade da ciência* congrega os trabalhos nos quais Kuhn tentou melhor estruturar, orgânica e sistematicamente, a nova abordagem sobre a constituição da ciência. Entretanto, a morte do autor impossibilitou a elaboração do formato final das ideias defendidas por ele. Contudo, a obra inacabada, mesmo se finalizada, dificilmente teria repercussão semelhante à de *A estrutura*. A erudição e o refinamento do livro não terminado o impediriam de alcançar a mesma popularidade popularidade. *A estrutura* atingiu marcas surpreendentes principalmente em função do convidativo didatismo utilizado pelo autor.

*A incomensurabilidade na ciência* concede bela oportunidade a profissionais ligados à história, à filosofia e à sociologia da ciência de desenvolverem visão mais qualificada das contribuições dadas por Kuhn. No panteão dos autores que moldaram o pensamento contemporâneo, a atuação de Kuhn não está restrita ao entendimento da produção do conhecimento. Todos os livros do autor permitem o aprimoramento da compreensão das complexidades do mundo atual, ancorada na importância dada à ciência em todas as esferas da vida em sociedade.

## Data Availability

Não estão em repositório.
